# MAGNET: Multi-view graph autoencoder with cell-gene attention for cell interaction network reconstruction from spatial transcriptomics

**DOI:** 10.1371/journal.pcbi.1013810

**Published:** 2025-12-15

**Authors:** Chengxu Han, Zhangdi Song, Zimu Xu, Jiaxing Chen

**Affiliations:** 1 Guangdong Provincial/Zhuhai Key Laboratory IRADS, Beijing Normal University–Hong Kong Baptist University United International College, Zhuhai, China; 2 Department of Computer Science, Hong Kong Baptist University, Hong Kong SAR, China; 3 Institute of Computer Science, University of Bern, Bern, Switzerland; 4 Key Laboratory for Artificial Intelligence and Multi-Model Data Processing, Department of Education of Guangdong Province, Zhuhai, China; Shanghai Institute of Nutrition and Health, Chinese Academy of Sciences, CHINA

## Abstract

Accurately inferring cell-cell interactions from spatial transcriptomics data remains challenging due to tissue complexity and spatial heterogeneity. Recent deep learning models have started to combine different types of cellular information, such as spatial proximity, gene expression similarity, ligand–receptor signaling, and in some cases, gene regulatory networks. However, they often treat a cell’s external interactions and internal gene regulation separately, merging them at the final step using simple concatenation or addition. This limits the model’s ability to capture how cell–cell communication and internal molecular states are connected. In this paper, we present MAGNET (Multi-view Graph Autoencoder with Cell-Gene Attention Network), a framework that reconstructs cell–cell interaction networks by building multiple biological graphs and developing a Cell-Gene attention module to link a cell’s environment with its internal gene activity in a unified representation. On benchmark datasets (seqFISH, MERFISH, STARMAP), MAGNET demonstrates superior performance in reconstructing cell-cell interaction networks, achieving an Average Precision(AP) of 0.901 on the seqFISH dataset and outperforming TENET by 0.185. The Cell-Gene Attention module is critical to MAGNET’s performance, as its removal alone caused the AP on the seqFISH dataset to drop from 0.901 to 0.521. Applied to a breast cancer dataset, MAGNET found functional heterogeneity among cancer cells, distinguishing clusters with molecular signatures for either immune evasion or autonomous tumor growth.

## Author summary

Intercellular interaction is a fundamental biological process that governs tissue function and is frequently dysregulated in diseases. While spatial transcriptomics technologies provide unprecedented data on cellular organization, accurately inferring the underlying interaction networks remains a significant challenge. Existing computational models oversimplify this complexity by prioritizing spatial proximity at the expense of other critical biological information. To address this, we developed MAGNET, a method designed to reconstruct cell-cell interactions by capturing a more comprehensive set of cellular relationships. By constructing a multi-view graph, MAGNET incorporates diverse biological signals in addition to spatial proximity, which are then integrate by a novel Cell-Gene attention module for a more accurate prediction. We show that this integrated approach enables MAGNET to perform well compared to existing methods. In breast cancer tissues, MAGNET identified diverse signaling networks, including those related to immune responses and extracellular matrix remodeling. In summary, MAGNET helps interpret intercellular signaling by combining different sources of cell–cell interaction information with gene expression data.

## Introduction

Cell-cell interactions (CCI) play a fundamental role in the regulation of critical biological processes such as tissue development, immune responses, and disease progression [1,2]. Understanding these interactions is essential for deciphering cellular communication and functional coordination, yet accurately constructing CCI remains challenging due to molecular complexity and spatial distribution heterogeneity [3]. Spatial Transcriptomics, which integrates gene expression data with spatial context, offers a promising strategy to resolve these intricate networks [4].

Although methods have been developed to detect CCI directly from spatial transcriptomic data, most existing methods such as CellPhoneDB, stLearn, NCEM , SpaCCC, GiottoTWCOM, CellChat [5–11] focus on interactions at the cell-type or cluster level, which neglects single-cell resolution and missing intratype cellular heterogeneity.

While COMMOT can reconstruct cell–cell communication networks at single-cell resolution, it primarily detects spatial factors and requires additional pathway information, which limits its ability [12]. NICHES visualizes heterogeneous signaling archetypes within cell groups, reflecting the local cellular microenvironment, but collapses cells into neighborhoods via principal component analysis, which may reduce noise, but lacks resolution at the single-cell level [13]. Due to limited spatial resolution and reliance on prior knowledge, most methods lack access to the full spectrum of cell information.

Recent graph-based deep learning methods have single-cell resolution interaction reconstruction. However, they mostly use spatial information to infer CCI and neglect key biological factors, such as ligand-receptor signaling, gene expression similarity, and gene network [14]. For example, DeepLinc focuses mainly on spatial distance in adjacency matrix preparation [15]. Clarify, employs the k-nearest neighbor algorithm, effectively capturing local spatial context but neglecting ligand-receptor signaling [16]. Similarly, TENET integrates Hierarchical Navigable Small World (HNSW) and Louvain modularity optimization to enhance spatially-driven community detection, yet it falls short in detecting transcriptionally similar but spatially distant cellular interactions [17]. A recent method CellNEST integrates ligand–receptor signaling with spatial context to model cell–cell communication [18] but do not consider gene network. Another limitation of the current methods is their use of pre-defined fusion strategies for integrating single-cell features. Although Clarify and TENET consider both cell-level and gene-level information in GNN, they implement feature fusion through simple strategies, such as concatenation or summation. OrgaCCC similarly integrates the two modalities via orthogonal graph autoencoders with a posterior loss-based coupling strategy, but lacks explicit intermediate-layer interactions [19]. These fusion approaches lack explicit architectural mechanisms to guide cross-modal feature interactions, placing the full burden of discovering these relationships on the message-passing mechanism. This limitation reveals a key need for a learnable fusion mechanism that can adaptively integrate cross-modal features in a biologically informed manner[20].

To address the limitation of existing methods, we propose MAGNET (Multi-view Graph Autoencoder with Cell–Gene Attention Network) to integrate gene networks and cell networks by capturing diverse aspects of cellular relationships. MAGNET incorporates multiple views of biological networks, such as spatial location, gene expression similarity, ligand–receptor interactions, and gene regulatory networks, to provide a comprehensive understanding of CCI.

## Methodology

**Fig 1:**
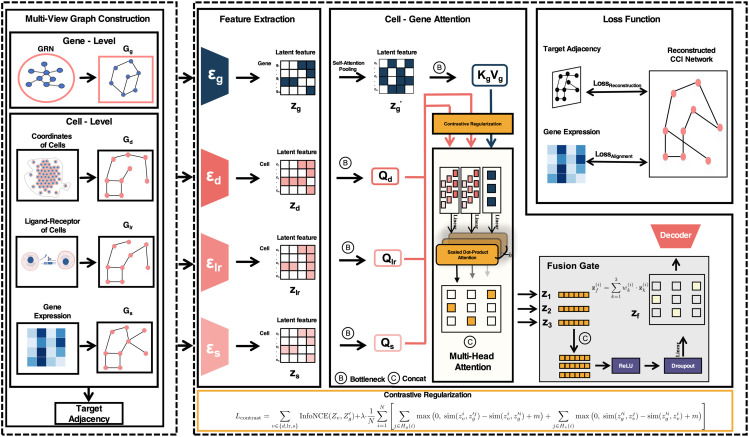
Overview of the multi-view graph autoencoder with cell-gene attention network.

As illustrated in Fig 1, MAGNET is a framework for reconstructing cell–cell interaction (CCI) networks from spatial transcriptomics data. It first constructs multiple graphs to capture gene-level and cell-level relationships from diverse biological views. Graph neural networks are applied to extract latent representations of genes and cells. These features are integrated through a Cell–Gene attention module that models fine-grained interactions between cells and genes. The fused representations are used to reconstruct the CCI network, guided by reconstruction and alignment loss functions.

### Multi-view graph construction

To comprehensively model potential cell–cell interactions at single-cell resolution, we construct four specific graphs from spatial transcriptomics data, namely a spatial proximity graph *G*_*d*_, a ligand–receptor interaction graph *G*_*lr*_, a transcriptional similarity graph *G*_*s*_, and a gene-level regulatory graph *G*_*g*_.

The spatial graph $G_{d}=(A_{d},X_{c})$ captures physical proximity between cells. The node feature matrix $X_{c}\in {\mathbb{R}}^{N\times G}$ represents the gene expression profiles of all cells, where each row corresponds to a cell and each column to a gene. Spatial relationships are encoded by the weighted adjacency matrix $W^{d}\in {\mathbb{R}}^{N\times N}$, where each entry is computed using an adaptive Gaussian kernel:

$$W_{ij}^{d}=\exp\left(-\frac{d_{ij}^{2}}{2\sigma_{i}^{2}}\right)$$ (1)

Here, *d*_*ij*_ is the Euclidean distance between cells *i* and *j*, and $\sigma_{i}$ is an adaptive bandwidth defined as the distance from cell *i* to its nearest neighbor *m*. The adaptive kernel modulates spatial influence by setting the bandwidth $\sigma_{i}$ according to local density. In dense regions, a small $\sigma_{i}$ leads to sharply localized affinities, whereas in sparse regions, a larger $\sigma_{i}$ allows the kernel to capture longer-range interactions, reflecting spatial influence between cells [21]. The binary adjacency matrix *A*_*d*_ is obtained by retaining the top *k* (k=5)most spatially relevant neighbors for each cell in *W^d^*, excluding self-loops.

The ligand–receptor graph $G_{lr}=(A_{lr},X_{c})$ is constructed to models ligand–receptor interactions, where the weighted adjacency matrix reflects interaction potentials between cell pairs.:

$$W_{ij}^{lr}=E_{l}^{\prime }(i)\cdot E_{r}^{\prime }(j)\cdot \exp\left(-\frac{d_{ij}^{2}}{2\sigma^{2}}\right)$$ (2)

where $E_{l}^{\prime }(i)$ and $E_{r}^{\prime }(j)$ represent the normalized expression of ligand and receptor in cells *i* and *j*. For each cell pair, interaction scores are computed across all ligand–receptor pairs and filtered using permutation testing (*p* < 0.05) to identify statistically significant interactions. The final edge weight is obtained by aggregating the scores of all significant ligand–receptor pairs for that cell pair. The adjacency matrix *A*_*lr*_ is constructed by sparsifying the interaction matrix *W^lr^*, retaining the top *k* (k=5) strongest outgoing edges per cell.

The transcriptional similarity graph $G_{s}=(A_{s},X_{c})$ captures transcriptomic similarity between cells. To construct the adjacency matrix *A*_*s*_, gene expression profiles are first normalized and reduced using PCA, producing an auxiliary embedding matrix $X_{s}\in {\mathbb{R}}^{N\times 32}$ used only for computing intercell distances. Pairwise Euclidean distances in this space are computed as:

$$d_{ij}=\|X_{s}^{\left(i\right)}-X_{s}^{\left(j\right)}{\|}_{2}$$ (3)

Local density is modeled using two parameters for each cell, where $\rho_{i}$ is the distance to its nearest neighbor and $\sigma_{i}$ is the distance to its *k*th nearest neighbor [22]. Based on these parameters, the conditional similarity from cell *i* to *j* is computed as

$$P(j|i)=\exp\left(-\frac{\max(0,d_{ij}-\rho_{i})}{\sigma_{i}+\epsilon }\right)$$ (4)

where ϵ is a small constant added for numerical stability. To obtain a symmetric similarity measure, the final similarity between cells *i* and *j* is given by

$$W_{ij}^{s}=P(j|i)+P(i|j)-P(j|i)\cdot P(i|j)$$ (5)

The adjacency matrix *A*_*s*_ is then constructed by retaining the top *k* (*k* = 5) most similar neighbors for each cell, excluding self-loops.

The gene-level graph $G_{g}=(A_{g},X_{g})$ models intracellular gene regulatory networks (GRNs), where nodes represent genes and edges indicate regulatory interactions. For each cell, a set of 30 genes was selected by prioritizing ligand–receptor pairs from curated databases (e.g. CellTalkDB) to ensure paired retention, and filling the remaining slots with the most highly expressed genes within the cell. The resulting expression matrix was normalized and reduced by principal component analysis (PCA) for covariance estimation. GRNs are inferred using CeSpGRN [23], producing block-diagonal adjacency matrices *A*_*g*_, each block corresponding to a cell-specific GRN. This structure captures regulatory patterns within individual cellular contexts while maintaining sparsity. Ligand–receptor annotations from curated databases [24] guide interaction directionality, ensuring biological plausibility. To extract latent topological features, Node2Vec [25] is applied to *G*_*g*_, generating the node feature matrix $X_{g}\in {\mathbb{R}}^{M\times d}$ where M=N×k denotes the total number of gene nodes, and N is the number of cells and k (k=30) is the number of selected genes per cell. The embedding dimension d is set to 64. These embeddings encode both local and global regulatory structures, enhancing gene-level interaction representations.

To guide the multi-view integration process, we construct a consensus cell–cell target adjacency matrix *A*_*target*_ using Similarity Network Fusion (SNF) [26]. This matrix combines three cell-level similarity measures into a unified representation of intercellular relationships. Each similarity matrix is first sparsified and normalized, then iteratively fused through cross-view message passing. The resulting matrix is thresholded to produce a binary adjacency matrix *A*_*target*_, which serves as a high-confidence supervisory target in the loss function. It is used only for training supervision and not as an input graph to the model.

### Multi-view feature extraction

To capture different aspects of cellular interactions, we construct four graph-based views and extract features from each using dedicated two-layer Graph Convolutional Networks (GCN) [27]. Three cell-level graphs, namely the spatial graph *G*_*d*_, the ligand-receptor graph *G*_*lr*_, and the transcriptional similarity graph *G*_*s*_, share the same set of nodes (cells) but differ in edge definitions that reflect spatial proximity, signaling potential, and transcriptional similarity. The input features for these graphs are the gene expression profiles $X_{c}\in {\mathbb{R}}^{N\times G}$, where *N* is the number of cells and *G* is the number of genes. The gene-level graph *G*_*g*_ models gene–gene relationships in cell, with input features $X_{g}\in {\mathbb{R}}^{M\times 64}$, where M denotes the number of expressed genes in that cell.

For each graph we let $Z^{\left(0\right)}=X_{c}$ (cell graphs) or $Z^{\left(0\right)}=X_{g}$ (gene graph) and perform feature extraction with the standard GCN propagation rule

$$Z^{\left(l+1\right)}=\sigma \left({\tilde{D}}^{-1/2}\tilde{A}{\tilde{D}}^{-1/2}Z^{\left(l\right)}W^{\left(l\right)}\right),$$ (6)

where $\tilde{A}$ is the adjacency matrix with self-loops, $\tilde{D}$ is its degree matrix, and *W^(l)^* is the trainable weight matrix. The two-layer GCN follows a bottleneck architecture. The first layer projects input features into a higher dimensional space, and the second maps them into a shared 32-dimensional latent space. This encourages the model to learn compact and informative representations [28].

To align the gene-level representation with cell-level features, we apply self-attention pooling over the structured gene embedding of each cell [29]. The input graph *G*_*g*_ is processed by a GCN to produce gene embeddings $Z_{g}\in {\mathbb{R}}^{M\times 32}$, which are then reshaped into $E_{g}\in {\mathbb{R}}^{N\times k\times 32}$.

Attention scores are computed within each cell using a shared two-layer MLP with a non-linear activation [29]. The resulting scalars are then normalised by a softmax over the *k* genes of that cell to produce attention weights $\beta_{ij}$ satisfying $\sum_{j=1}^{k}\beta_{ij}=1$.

The cell-specific gene representation is then

$$Z_{g}^{\prime }(i)=\sum_{j=1}^{k}\beta_{ij}\cdot e_{ij}.$$ (7)

producing the matrix $Z_{g}^{\prime }\in {\mathbb{R}}^{N\times 32}$ that captures local regulatory context and is dimensionally compatible with other cell-level embeddings.

As a result we obtain four embedding sets: three cell-level embeddings $(Z_{d},Z_{lr},Z_{s})\in {\mathbb{R}}^{N\times 32}$ and the gene-level embedding $Z_{g}^{\prime }\in {\mathbb{R}}^{N\times 32}$.

### Contrastive regularization

To ensure integration across modalities, we introduce a contrastive regularization step. This step aligns each of the three cell-view embeddings $Z_{d},Z_{lr},Z_{s}$ with the shared gene-level embedding $Z_{g}^{\prime }$.

To achieve this, we formulate the contrastive loss $L_{contrast}$ as the sum of two complementary components. The first component is the standard symmetric InfoNCE loss [30]. It performs a global alignment by treating all non-matching samples within a batch as negatives, encouraging the model to distinguish the correct positive pair from a diverse set of distractors:

$$InfoNCE(Z_{v},Z_{g}^{\prime })=\frac{1}{2N}\sum_{i=1}^{N}[-\log\frac{\exp\left(\frac{\mathrm{sim}(z_{v}^{i},{z_{g}^{\prime }}^{i})}{\tau }\right)}{\sum_{j=1}^{N}\exp\left(\frac{\mathrm{sim}(z_{v}^{i},{z_{g}^{\prime }}^{j})}{\tau }\right)}\;-\log\frac{\exp\left(\frac{\mathrm{sim}({z_{g}^{\prime }}^{i},z_{v}^{i})}{\tau }\right)}{\sum_{j=1}^{N}\exp\left(\frac{\mathrm{sim}({z_{g}^{\prime }}^{i},z_{v}^{j})}{\tau }\right)}]$$ (8)

where all embeddings are L2-normalized, and similarities are computed using the dot product scaled by a temperature parameter τ=0.3. While InfoNCE is effective, its efficiency may decline as the model quickly learns to ignore negative samples that are easily separable from the anchor [31].

To improve training dynamics and enhance the model’s ability to distinguish between similar cases, we introduce a margin-based contrastive loss. Let $Z_{g}^{\prime }\in {\mathbb{R}}^{N\times 32}$ denote the aligned gene-level embeddings, where each row corresponds to a representative gene feature assigned to cell *i*. We denote *H*_*g*_(*i*) ⊂ {1,…,N} ⧵ {*i*} as the indices of non-matching gene embeddings used as hard negatives for the cell anchor $z_{v}^{i}$. Similarly, let $H_{v}(i)$ ⊂ {1,…,N} ⧵ {*i*} denote the indices of non-matching cells used as hard negatives for the gene anchor $z_{g}^{\prime \;i}$, selected from the view-specific embeddings $Z_{v}$. The margin-based loss for a single view *v* is defined as:

$$L_{margin}=\frac{1}{N}\sum_{i=1}^{N}[\sum_{j\in H_{g}(i)}\max(0,\;\mathrm{sim}(z_{v}^{i},z_{g}^{\prime \;j})-\mathrm{sim}(z_{v}^{i},z_{g}^{\prime \;i})+m)\;\;+\sum_{j\in H_{v}(i)}\max(0,\;\mathrm{sim}(z_{g}^{\prime \;i},z_{v}^{j})-\mathrm{sim}(z_{g}^{\prime \;i},z_{v}^{i})+m)]$$ (9)

We fix the margin hyper-parameter at *m* = 0.1 for all experiments.

The overall contrastive loss combines the symmetric InfoNCE term and the margin-based term, weighted by a hyperparameter λ (λ=0.1):

$$L_{contrast}=\sum_{v\in \{d,lr,s\}}\left(InfoNCE(Z_{v},Z_{g}^{\prime })+\lambda \cdot L_{margin}(Z_{v},Z_{g}^{\prime })\right)$$ (10)

This combined regularization encourages consistent representations, while focusing the model on distinctions, thus providing a foundation for the downstream Cell-Gene Attention fusion module.

### Cell-gene attention

Cell-Gene Attention integrates cell-level and gene-level representations based on multiple biological views. We retain three distinct cell representations: the spatial proximity view $Z_{d}\in {\mathbb{R}}^{N\times 32}$, the ligand-receptor interaction view $Z_{lr}\in {\mathbb{R}}^{N\times 32}$, and the transcriptional similarity view $Z_{s}\in {\mathbb{R}}^{N\times 32}$. Each view is independently derived using a dedicated GCN, allowing the model to capture complementary information from different aspects of cellular organization. For gene-level input, we use the aligned embedding $Z_{g}^{\prime }\in {\mathbb{R}}^{N\times 32}$, obtained via attention pooling over cell-specific gene subgraphs.

Cross multihead attention is computed in three parallel branches. Each cell view, spatial (*Z*_*d*_), ligand-receptor (*Z*_*lr*_), and transcriptional (*Z*_*s*_), serves as the query input to its respective attention branch, while the shared gene embeddings $Z_{g}^{\prime }\in {\mathbb{R}}^{N\times d}$ are used as keys and values across all branches. For a given attention head *h* , the computation is:

$$Q_{h}=Z_{c}W_{q}^{h},\;K_{h}=Z_{g}^{\prime }W_{k}^{h},\;V_{h}=Z_{g}^{\prime }W_{v}^{h},$$ (11)

where $Z_{c}\in {\mathbb{R}}^{N\times d}$ denotes one of the three cell views, and $W_{q}^{h},W_{k}^{h},W_{v}^{h}\in {\mathbb{R}}^{d\times d_{k}}$ are trainable projection matrices for the *h*th head. We use *H* = 4 heads and set *d*_*k*_ = *d*/*H*.

Attention weights are computed using scaled dot-product attention, and each head output is aggregated:

$$\alpha_{ij}^{h}=\frac{\exp\left(Q_{h}(i{)}^{⊤}K_{h}(j)/\sqrt{d_{k}}\right)}{\sum_{j^{\prime }=1}^{N}\exp\left(Q_{h}(i{)}^{⊤}K_{h}(j^{\prime })/\sqrt{d_{k}}\right)},\;O_{h}(i)=\sum_{j=1}^{N}\alpha_{ij}^{h}V_{h}(j).$$ (12)

Outputs from all *H* heads are concatenated and projected through a shared matrix $W_{o}\in {\mathbb{R}}^{Hd_{k}\times d}$, yielding three fused representations: $Z_{f1},Z_{f2},Z_{f3}\in {\mathbb{R}}^{N\times d}$, each corresponding to one of the three branches.

Each output is subsequently refined through a residual connection, a layer-normalization layer, and a position-wise feed-forward network (FFN). The FFN applies two linear transformations with an intermediate GELU nonlinearity:

$$FFN(x)=W_{2}\;GELU(W_{1}x+b_{1})+b_{2},$$ (13)

where $W_{1}\in {\mathbb{R}}^{4d\times d}$, $W_{2}\in {\mathbb{R}}^{d\times 4d}$, and *b*_1_, *b*_2_ are bias terms. A second residual connection after the FFN preserves representational stability.

Finally, to integrate the three fused outputs, we employ a gated fusion mechanism. A lightweight two-layer MLP takes the concatenated outputs Concat$\left(Z_{f1},Z_{f2},Z_{f3}\right)$ and produces dynamic weights $\left(w_{1},w_{2},w_{3}\right)$ via softmax [32]. The final embedding is computed as:

$$Z_{final}=\sum_{k=1}^{3}w_{k}\cdot Z_{fk}.$$ (14)

This design enables the model to dynamically emphasize the most informative view for each cell, enhancing robustness and representational capacity.

### Model optimization and loss functions

MAGNET is trained to learn the final cell embeddings $Z_{final}\in {\mathbb{R}}^{N\times 32}$, which are optimized to reconstruct a target adjacency matrix $A_{target}\in \{0,1{\}}^{N\times N}$. A standard inner product decoder is used to compute the predicted adjacency matrix:

$${\hat{A}}_{ij}=\sigma (Z_{final,i}\cdot Z_{final,j}^{⊤}),$$ (15)

where σ(·) denotes the sigmoid activation. The final and primary reconstruction loss is a Binary Cross-Entropy (BCE) loss [33], designed to approximate the target adjacency matrix:

$$L_{recon}=-\frac{1}{N^{2}}\sum_{i,j}\left[A_{target,ij}\log({\hat{A}}_{ij})+(1-A_{target,ij})\log(1-{\hat{A}}_{ij})\right],$$ (16)

In addition to this primary objective, we include two lightweight regularization terms. The first is a contrastive loss $L_{\_contrast}$, applied before fusion to align cell-view and gene-level embeddings. The second regularization term is a Center Alignment Loss*L*_*alignment*_, which encourages the final embeddings to remain close to a projected mean expression anchor:

$$L_{alignment}=\frac{1}{N}\sum_{i=1}^{N}{\left\|Z_{4,i}-P({\bar{X}}_{c})\right\|}_{2}^{2},\;{\bar{X}}_{c}=\frac{1}{N}\sum_{i=1}^{N}X_{c,i}.$$ (17)

The total training objective is defined as:

$$L_{\text{total}}=L_{recon}+0.1\cdot L_{\text{contrast}}+0.1\cdot L_{alignment}.$$ (18)

This formulation ensures that the final embeddings are primarily optimized for interaction prediction, while remaining stable and well-aligned across biological modalities.

### Experimental design and model evaluation

We benchmarked MAGNET on three publicly available spatial transcriptomics datasets: seqFISH (1,597 cells, 125 genes) [34], MERFISH (2,000 cells, 160 genes) [35], and STARMAP (1,207 cells, 257 genes) [36]. To evaluate model performance under different supervision signals, we used two types of target adjacency matrices: a standard Distance-Based adjacency derived from spatial proximity, and a Multi-View adjacency constructed via Similarity Network Fusion by integrating spatial, ligand–receptor, and transcriptional similarities. To further investigate the trade-off between dataset size and computational efficiency, we performed additional scalability experiments on the MERFISH dataset, as shown in S2 Fig.

MAGNET was compared against four recent deep learning models for CCI inference. DeepLinc uses a Variational Graph Autoencoder (VGAE) to model proximity-based graphs. Clarify integrates gene-level and cell-level information through GCN to enhance representation learning. TENET improves interaction prediction by denoising spatial expression data and optimizing deep graph architectures. CellNest, the most recent among them, adopts an unsupervised framework that integrates spatial coordinates and ligand–receptor priors to learn latent cell representations. As it does not rely on an explicit graph reconstruction objective, we evaluated its performance by using the learned embeddings for downstream interaction prediction, in line with the setup used for other models. All models were trained for up to 120 epochs with early stopping, and we report the mean and standard deviation of AP and AUC, averaged over 10 runs with different random seeds. An edge-level split strategy was used, where positive edges were randomly sampled from the target adjacency matrix and negative edges were randomly selected from non-connected pairs. These were divided into training, validation, and test sets. This setting follows the evaluation protocols of CLARIFY, DeepLinc, and TENET to ensure a fair and consistent comparison.

To further understand the internal mechanisms and design choices of MAGNET, we conducted two sets of ablation experiments. The first assessed the contribution of three architectural components: the Cell Gene Attention mechanism for multimodal fusion, the Contrastive Regularization for aligning loss function. This analysis helped reveal which modules most strongly influence overall performance. The second ablation focused on the input graph modalities by evaluating the model using only one pair of graphs at a time, namely spatial proximity with transcriptional similarity, spatial proximity with ligand receptor interactions, and transcriptional similarity with ligand receptor interactions. This design allowed us to assess both the necessity of multi view integration and the informativeness of each individual view.

Finally, we demonstrate MAGNET’s applicability to datasets with broader transcriptomic coverage in a case study using 10x Visium data. Specifically, we evaluate the model on two samples: a human breast cancer section (4,000 cells, 200 genes) and a mouse brain section (3,597 cells, 200 genes). Gene selection was performed by retaining highly variable genes that are non-zero across all cells. Since official annotations of cell cluster types are not available for these datasets, we performed cluster-level labeling using external references. For the human breast cancer sample, we applied Seurat to identify transcriptionally distinct clusters [37], and annotated each cluster as either cancerous or non-cancerous based on known biomarker gene expression patterns. For the mouse brain sample, we assigned anatomical labels to clusters by aligning spatial locations with the Allen Mouse Brain Atlas [38], using region-specific landmarks to distinguish major brain structures such as cortical layers, hippocampus, and subcortical regions. The MAGNET method and trained models have been released as open-source resources and are available at https://github.com/FrankBNBU/MAGNET.

### Downstream analysis and biological validation

To assess the biological relevance of MAGNET’s predictions, we conducted a structured downstream analysis focused on the top 5% of high-confidence cell–cell interactions. This analysis encompassed spatial and topological validation, functional enrichment, and mechanistic inference.

In network of spatial structure, we evaluated spatial coherence by comparing the Euclidean distance distributions of intra cluster and inter cluster interacting cell pairs. Differences were assessed using cumulative distribution plots and the Kolmogorov–Smirnov test [39]. Additionally, we analyzed network topology via node degree and closeness centrality to identify hub cells and characterize global connectivity [40].

For functional validation, we analyzed the most frequent ligand–receptor pairs mediating the predicted interactions. Gene Ontology (GO) and KEGG enrichment analyses were performed to determine whether these interactions are associated with biologically meaningful signaling pathways relevant to the tissue context.

To explore underlying biological mechanisms, we used FlowSig [41] to infer active gene expression modules (GEMs) for each cell, providing a compact representation of intracellular functional state. To characterize cluster-level functional responses, we examined receiver clusters that were frequently targeted in the reconstructed interaction network. For each such cluster, we identified GEMs that were repeatedly involved in incoming interactions and performed KEGG enrichment to infer the dominant processes likely mediating their signal reception.

## Results

We evaluated MAGNET’s performance in CCI networks and its utility for biological interpretation through a comprehensive set of analyses. First, we assessed its accuracy by comparing it with recent deep learning models, including TENET, DeepLinc, CellNEST, and Clarify, on three publicly available spatial transcriptomics datasets. Second, we conducted ablation studies to investigate the contributions of key architectural components and input modalities to overall performance. Third, we applied MAGNET to human breast cancer and mouse brain datasets to evaluate its capacity to support biological discovery. In these case studies, we analyzed high-confidence interactions in terms of their spatial organization, network topology, and associated cellular functions, using gene module aggregation and pathway enrichment analysis. Together, these results demonstrate that MAGNET not only achieves accurate reconstruction of CCI networks but also provides insights into their spatial and functional organization across diverse biological contexts.

### Model performance comparison

MAGNET outperformed baseline models in reconstructing CCI networks across multiple spatial transcriptomics datasets, demonstrating generalizability and robustness.

First, comparative results across models, datasets, and data split ratios are summarized in Table 1. MAGNET achieved the highest AP scores across all datasets and splits, with advantages under limited-data conditions. For example, it reached AP scores of 0.926 and 0.870 at splits of 0.7 and 0.9 on MERFISH (Distance-Based target adjacency), and 0.872 at split 0.9 on seqFISH (Multi-View target adjacency). Similar performance was observed on STARmap (Multi-View), with APs of 0.897, 0.846, and 0.823 at splits of 0.5, 0.7, and 0.9, respectively. Baseline models exhibited varied behaviors under different conditions. CellNEST performed well with abundant training data but deteriorated significantly in sparse or multi-view settings. TENET showed competitive results on Distance-Based targets but declined sharply under Multi-View evaluations. DeepLinc and Clarify yielded relatively stable yet consistently lower scores across all conditions.

**Table 1:** Performance comparison of MAGNET and baseline models on MERFISH, seqFISH, and STARmap datasets under Multi-View target adjacency matrix.

Method	MERFISH				seqFISH				STARmap			
	split0.3	split0.5	split0.7	split0.9	split0.3	split0.5	split0.7	split0.9	split0.3	split0.5	split0.7	split0.9
**DeepLinc**	0.857	0.812	0.734	0.632	0.707	0.700	0.666	0.595	0.826	0.784	0.723	0.588
**Clarify**	0.846	0.793	0.701	0.607	0.880	0.830	0.740	0.630	0.871	0.806	0.726	0.614
**TENET**	0.822	0.768	0.649	0.570	0.918	0.761	0.715	0.589	0.787	0.795	0.713	0.574
**MAGNET**	**0.932**	**0.924**	**0.906**	**0.888**	**0.949**	**0.937**	**0.901**	**0.872**	**0.901**	**0.897**	**0.846**	**0.823**
**CellNEST**	0.721	0.695	0.648	0.570	0.714	0.658	0.623	0.531	0.763	0.756	0.661	0.581

In addition to the multi-view comparison, we further validated MAGNET on individual single-view graphs to examine its robustness across different types of cellular relationships. As summarized in Table 2, MAGNET generally achieved superior or comparable performance to the baselines across the three single-view settings, including distance-based, ligand–receptor, and transcriptional similarity graphs, indicating that the model effectively leverages diverse biological priors and maintains stable generalization across distinct graph modalities.

**Table 2:** Performance comparison of MAGNET and baseline models on three spatial transcriptomics datasets (seqFISH, MERFISH, and STARmap) using single-view target adjacency matrices at split 0.3.

Method	Distance-based			Ligand–receptor (LR)			Transcriptional similarity		
	seqFISH	MERFISH	STARmap	seqFISH	MERFISH	STARmap	seqFISH	MERFISH	STARmap
DeepLinc	0.746	0.723	0.824	0.928	0.897	0.854	0.778	0.872	0.650
Clarify	0.910	0.905	0.674	0.853	0.807	0.812	0.788	0.889	0.716
TENET	0.933	0.925	0.627	0.895	0.892	0.771	**0.879**	0.778	0.754
MAGNET	**0.948**	0.935	0.880	**0.952**	**0.949**	**0.931**	0.856	**0.919**	**0.849**
CellNEST	0.910	**0.951**	**0.910**	0.826	0.724	0.793	0.860	0.850	0.823

**Fig 2:**
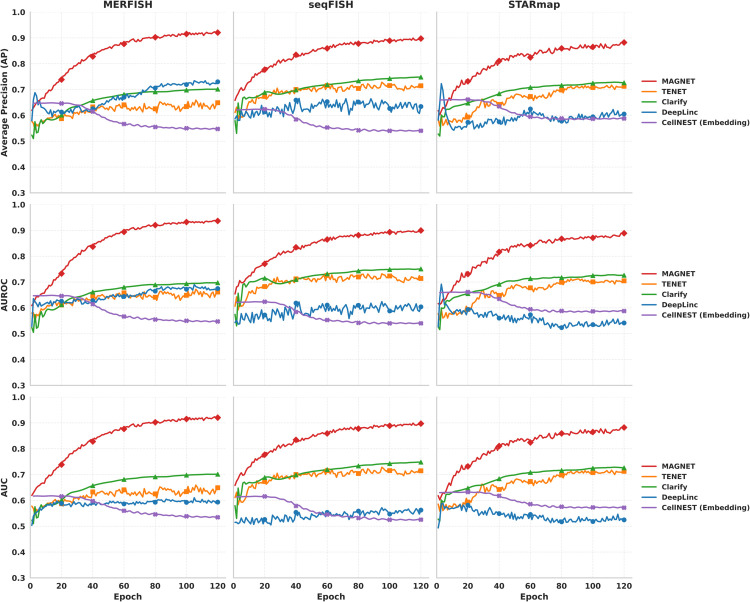
Training trends of different methods on three single-cell resolution spatial transcriptomics datasets (MERFISH, seqFISH, and STARmap), evaluated using Average Precision (AP), AUROC, and AUC over 120 training epochs.

Second, as shown in Fig 2, MAGNET outperformed all baselines throughout the training process across three spatial transcriptomics datasets. It achieved higher final scores in AP, Area Under the Receiver Operating Characteristic Curve (AUROC), and Area Under the Precision–Recall Curve (AUC). In addition, MAGNET exhibited greater training stability, with smaller fluctuations and smoother trajectories over 120 epochs.

**Fig 3:**
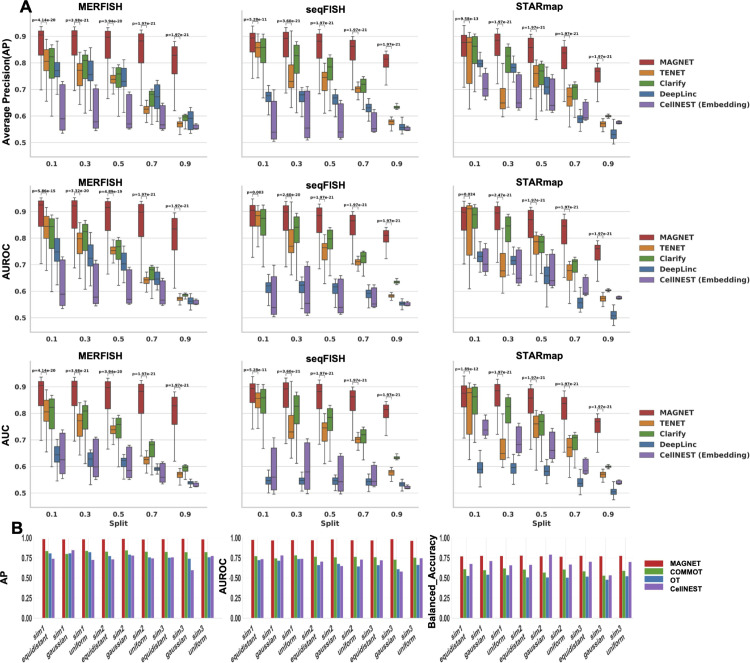
(A) Box plots summarizing the test performance of different models on three single-cell–resolution spatial transcriptomics datasets under the multi-view target adjacency matrix. Results across varying train–test split ratios demonstrate the robustness and consistency of MAGNET after 300 training epochs. Statistical significance between MAGNET and TENET was evaluated using p-value comparisons across all metrics. (B) Bar plots comparing MAGNET with other representative cell–cell communication models (COMMOT, OT, and CellNEST) on simulated spatial datasets from the COMMOT benchmark under the multi-view setting. The evaluation based on Average Precision (AP), AUROC, and Balanced Accuracy highlights MAGNET’s superior and stable performance across datasets.

Third, extended experiments with 300 training epochs (Fig 3) confirmed that MAGNET maintained the highest and most stable performance across datasets and metrics, exhibiting smaller performance drops and narrower variability as training data became increasingly scarce. Compared to TENET, MAGNET achieved superior results, with paired Wilcoxon signed-rank test p-values <0.05 across all three metrics. In addition to GNN-based baselines, we further compared MAGNET with two representative non-GNN CCI inference frameworks: COMMOT, which implements an optimal transport (OT) formulation, and CellNEST, an unsupervised method based on gene expression embedding. As shown in Fig 3B, benchmarking under three simulation settings and diverse data distributions demonstrated that MAGNET consistently achieved higher AP, AUROC, and balanced accuracy scores than both COMMOT and CellNEST, highlighting its reliability across heterogeneous conditions. Together, these results underscore MAGNET’s superior robustness, generalization, and interpretability in modeling complex intercellular communication networks.

To further evaluate the reconstructed networks, we compared MAGNET and SNF at both global and regional levels (see S1 Fig). For quantitative evaluation, we selected the top 20,000 edges from both networks and calculated the proportions of ligand–receptor (LR) related and high-expression supported edges. The LR edges were determined based on curated ligand–receptor gene pairs, and expression values were Z-score normalized across cells, with high-expression edges defined as those in which both ligand and receptor genes had Z-scores greater than 1 in the connected cells. Under these criteria, MAGNET reconstructed a higher proportion of ligand–receptor–related and high-expression–supported edges than SNF, indicating that MAGNET-derived networks capture a larger number of biologically supported interactions.

These results demonstrate that MAGNET captures complex multi-view cellular relationships and supports accurate and robust CCI network reconstruction across a range of spatial transcriptomics contexts.

### Ablation experiment

To comprehensively assess the contribution of each core component to MAGNET’s performance, we conducted two sets of ablation experiments. The first evaluated key architectural modules, and the second investigated the importance of different input graph modalities.

**Fig 4:**
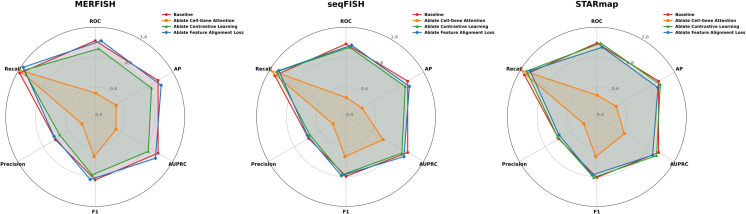
Performance impact of MAGNET’s core modules assessed via ablation studies. Each radar chart visualizes the model’s performance on a specific dataset after the removal of a key architectural component. The axes represent different evaluation metrics or datasets, and the area of the polygon indicates overall performance, demonstrating the critical contribution of each module.

The first set of ablations assessed the role of major components. As shown in Fig 4, radar plots summarize the performance of ablated variants across the MERFISH, seqFISH, and STARmap datasets, evaluated using six metrics: AP, AUROC, AUPRC, F1-score, Precision, and Recall. The full MAGNET model outperformed all ablations. Removing the Cell-Gene Attention module resulted in the most substantial performance drop across all metrics, underscoring its critical role in capturing interactions between cells and genes. Excluding the contrastive learning module led to moderate reductions in AUPRC and F1-score, indicating its contribution to improving representation quality and inter-modality consistency. Eliminating the feature alignment loss caused smaller but consistent performance declines, suggesting its role in stabilizing multi-view fusion. Together, these results highlight the importance of all three components, with Cell-Gene Attention being the most indispensable for MAGNET’s overall effectiveness.

The second ablation study evaluated the necessity of different input graph modalities: spatial proximity, gene similarity, and ligand–receptor interactions. Table 3 reports the AP, AUC, and ROC scores across various modality combinations. Using all three modalities consistently achieved the best performance. For example, on seqFISH, the complete combination reached an AP of 0.916, while performance dropped when any modality was excluded. Specifically, AP fell to 0.868 with spatial and gene similarity graphs, 0.786 with spatial and ligand–receptor graphs, and 0.804 with gene similarity and ligand–receptor graphs. Similar trends were observed on MERFISH and STARmap datasets. In addition, to ensure a fair comparison with single-view approaches, we included a setting that used only the spatial distance graph as input, identical to other baselines Table 3. The model still achieved better results under this condition, indicating that its improvement does not solely stem from shared information sources. These results highlight the complementary roles of all three graph types and confirm that their integration is essential for MAGNET to effectively model spatial transcriptomics data.

**Table 3:** Ablation study of graph type usage on different datasets.

Dataset	Graph Type			Performance		
	Spatial	LR	Gene	AP	AUC	ROC
seqFISH	✓			0.753	0.742	0.745
	✓	✓		0.868	0.864	0.870
	✓		✓	0.786	0.787	0.781
		✓	✓	0.804	0.792	0.792
	✓	✓	✓	**0.901**	**0.905**	**0.921**
MERFISH	✓			0.772	0.778	0.781
	✓	✓		0.888	0.889	0.893
	✓		✓	0.792	0.799	0.799
		✓	✓	0.807	0.795	0.794
	✓	✓	✓	**0.926**	**0.911**	**0.912**
STARmap	✓			0.738	0.734	0.732
	✓	✓		0.812	0.806	0.806
	✓		✓	0.779	0.773	0.772
		✓	✓	0.769	0.755	0.755
	✓	✓	✓	**0.853**	**0.836**	**0.842**

Together, these ablation studies establish that MAGNET’s performance arises from the synergistic integration of its architectural innovations and diverse graph inputs, each playing a crucial and complementary role in robust spatial transcriptomics analysis.

### Hyperparameter experiment

**Fig 5:**
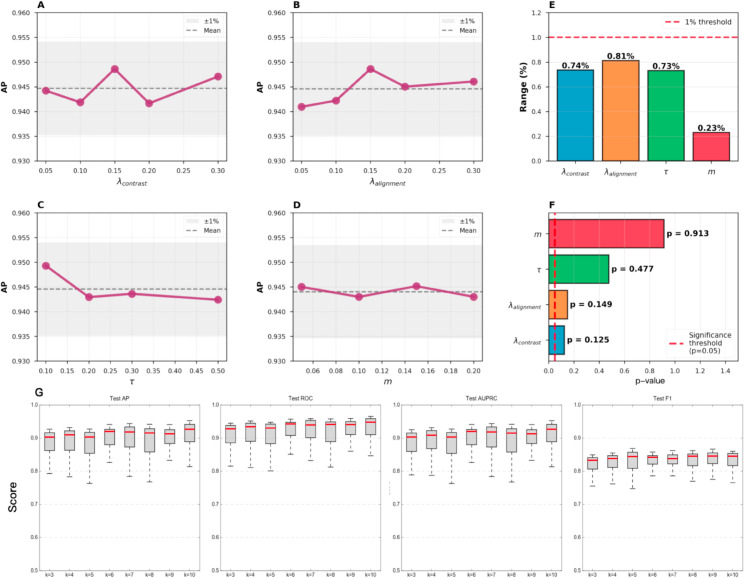
Hyperparameter sensitivity analysis of MAGNET. (A–D) Effects of $\lambda_{contrast}$, $\lambda_{alignment}$, *τ*, and *m* on average precision (AP). (E–F) Quantitative summaries showing the variation range (<1) and corresponding p-values for each parameter, all remaining above 0.05, indicating no statistically significant difference across settings. (G) Evaluation of the neighborhood size *k* (the number of nearest neighboring cells) in graph construction across spatial, ligand–receptor, and transcriptional graphs. MAGNET maintains stable AP, ROC-AUC, and F1 scores when *k* varies from 3 to 10, confirming robustness to local connectivity settings.

To evaluate the stability and robustness of MAGNET with respect to its key hyperparameters, we conducted a comprehensive sensitivity analysis covering both loss-related and structural parameters. The tested hyperparameters included the contrastive loss weight ($\lambda_{contrast}$), feature alignment loss weight ($\lambda_{alignment}$), temperature parameter (*τ*), attention margin (*m*), and the neighborhood size (*k*) used in graph construction (Fig 5).

As shown in Fig 5A–5D, the model’s average precision (AP) fluctuated within 1% across a broad range of loss coefficients and attention parameters, indicating that the optimization objectives are well balanced and not overly sensitive to tuning. Quantitative comparisons of parameter ranges and corresponding *p*-values (Fig 5E–5F) confirmed that none of these hyperparameters exerted statistically significant effects on final performance (*p* > 0.05). This suggests that the training process remains stable under moderate parameter variation.

We further examined the influence of the neighborhood size *k*, which defines local connectivity in the spatial, ligand–receptor, and transcriptional graphs (Fig 5G). When *k* was varied from 3 to 10, the model’s AP, ROC-AUC, and F1 scores remained nearly constant, demonstrating robustness across a wide range of spatial resolutions. This result also aligns with previous studies such as Clarify, DeepLinc, and TENET, which adopt *k* = 5 as a standard configuration for spatial transcriptomics graphs.

Overall, these analyses indicate that MAGNET is not highly sensitive to individual hyperparameter settings. The model achieves stable and reliable performance without tuning, supporting its practicality and reproducibility across datasets.

### Investigation of functional heterogeneity in breast cancer using MAGNET

**Fig 6:**
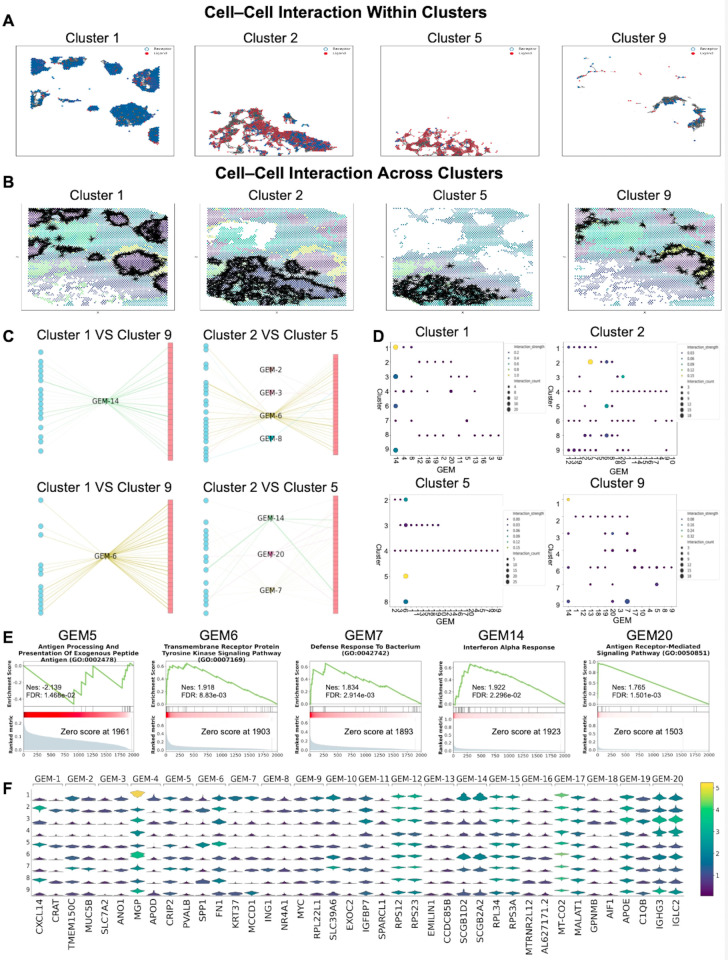
This Fig presents the MAGNET reconstructed cell-cell interaction network derived from spatial transcriptomic cancer data, highlighting potential roles in the tumor microenvironment. It visualizes ligand–receptor interactions within individual clusters(A), while highlighting functional interactions between different clusters (B), and provides detailed insights into interactions among specific cell clusters at the level of Gene Expression Modules (GEMs) (C). The analysis is based on GEMs, showing the number of times and the flow values with which different clusters are connected through each GEM (D). Functional insights are provided through Gene Set Enrichment Analysis (GSEA) of key biological pathways (E). (F) shows representative gene signatures for each GEM.

We applied MAGNET to spatial transcriptomics data from human breast cancer to investigate the functional heterogeneity of intercellular interactions within the tumor microenvironment. Reconstructed CCI networks within and between transcriptionally defined clusters are shown in Fig 6A–6B. Spatial domain analysis using Seurat [37] identified Clusters 5 and 9 as tumor-enriched regions, whereas Clusters 1 and 2 were transcriptionally distinct, non-malignant compartments adjacent to the tumor.

MAGNET uncovered distinct modes of communication across these regions, reflecting divergent transcriptional programs. GEM14 was identified as the dominant communication module mediating frequent interactions between Clusters 1 and 9 (Fig 6C–6D). This module was enriched for immune-related pathways such as antigen processing and interferon signaling (Fig 6E) and prominently featured SCGB1D2 and SCGB2A2 (Fig 6F), two secretoglobin family members previously associated with luminal-type epithelial cells and epithelial differentiation [42]. These results may suggest that GEM14 captures a localized epithelial–immune transcriptional program at the tumor–normal interface.

In contrast, GEM6 was identified as the main module connecting Clusters 2 and 5. Enrichment analysis showed that this module is strongly associated with transmembrane receptor protein tyrosine kinase signaling and extracellular matrix remodeling pathways (Fig 6E). It includes high expression of SPP1 and FN1 (Fig 6F), two genes that are recognized for their roles in promoting matrix remodeling and tumor invasion [43–47]. These results suggest the presence of a pro-invasive transcriptional program in a microenvironment that is rich in extracellular matrix and relatively lacking in immune activity.

Together, these findings show that MAGNET can help analyze the functional heterogeneity within tumor microenvironments by linking reconstructed CCI to their underlying gene expression programs. By identifying communication modules, MAGNET reveals spatially organized patterns of intercellular signaling, from immune-related activity at the tumor boundary to pro-invasive programs in extracellular matrix–rich regions. This demonstrates the utility of MAGNET for studying spatial variation in tumor biology.

#### MAGNET identifies cell–cell interaction networks in the mouse brain.

**Fig 7:**
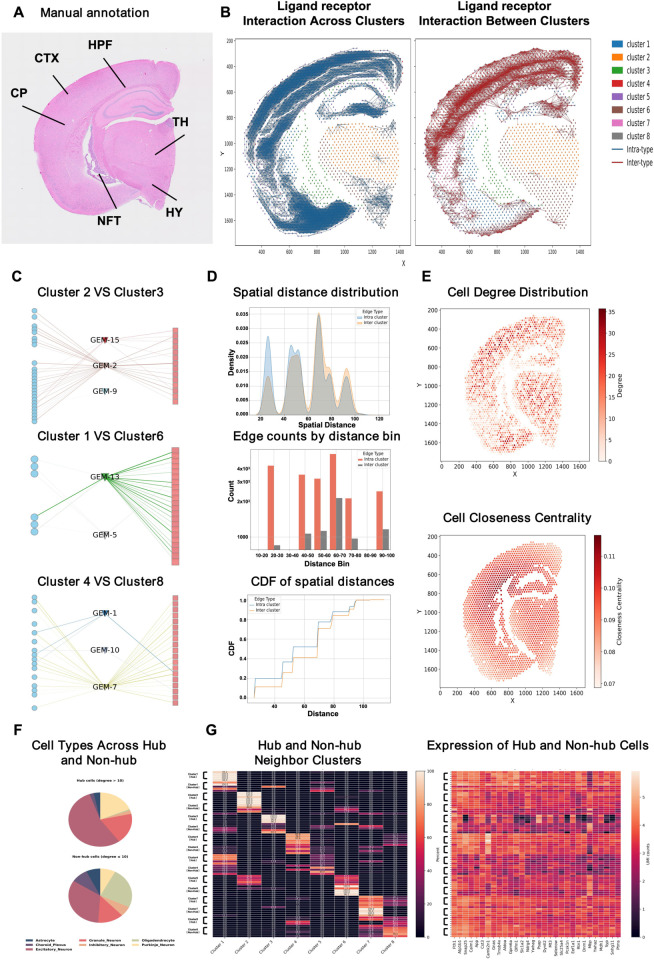
MAGNET reconstructs spatially organized intercellular communication in the mouse brain. (A) Manual annotation of major anatomical regions (CTX, HPF, CP, TH, HY, NFT). (B) Reconstructed ligand–receptor interaction networks showing intracluster (left) and intercluster (right) signaling patterns. (C) Spatial distance distribution of interaction edges. (D) Edge counts by distance bin and the cumulative distribution function (CDF) of distances. (E) Node-level topology: distributions of cell degree and closeness centrality, highlighting local communication hubs. (F) Cell-type composition of hubs (degree > 10) and non-hubs. (G) Per-cluster comparison of five hubs and five non-hubs. Left: for each cluster, we compare the distribution of linked neighbor clusters for hubs versus non-hubs. Right: for each cluster, we compare normalized gene-expression between hubs and non-hubs.

Spatial transcriptomics data from the mouse brain were used to assess how MAGNET infers intercellular communication. Annotation provided reference labels for major anatomical regions, including the cortex (CTX), thalamus (TH), and hippocampus (HPF), against which transcriptionally defined clusters were compared (Fig 7A). MAGNET revealed dense intra cluster signaling, while also identifying coherent inter cluster interactions across region boundaries (Fig 7B).

As shown in Fig 7C, MAGNET identified several GEMs that mediate communication between specific cluster pairs, each associated with spatially distinct brain regions. For example, GEM2 and GEM9 were predominantly active between Clusters 2 and 3, which are located in adjacent cortical zones, suggesting region-specific synaptic signaling. GEM5 mediated interactions between Clusters 1 and 6, which spatially bridge cortex and thalamus, potentially reflecting integration across functional areas.

We quantified the spatial properties of the reconstructed network (Fig 7D–7E). Intra cluster interactions were short-range, while inter cluster communication occurred over longer distances, consistent with physical tissue organization. Cells with high degree and closeness centrality were enriched at cluster boundaries, suggesting they serve as communication hubs bridging spatially distinct cell populations.

Finally, We defined cells with total interaction degree > 10 as hubs and, for each cluster, selected five hubs and five non-hubs for comparison. In terms of cell-type composition (Fig 7F), hubs are predominantly neuronal, mainly excitatory with a contribution from inhibitory neurons (and Purkinje neurons where cerebellar tissue is present), whereas non-hubs show a more mixed makeup with a higher oligodendrocyte fraction. In Fig 7G, hubs preferentially wire within their own clusters and preserve lineage-matched programs, whereas non-hubs connect more broadly across clusters and exhibit neighbor-aligned expression. Cluster-wise, Cluster 1 hubs stay within the excitatory network and maintain Ptms, Tmsb4x, Gnas; Cluster 1 non-hubs link to astrocytes and choroid. Cluster 2 hubs remain oligodendrocyte-centric yet express Snap25, Atp1b1, Slc25a4; Cluster 2 non-hubs are more purely oligodendrocytic. Cluster 3 hubs keep Psap, Mdh1, Gnas; Cluster 3 non-hubs connect to oligodendrocytes and excitatory cells and show Mbp and Pcsk1n. Across Cluster 4/Cluster 7/Cluster 8, hubs interconnect excitatory modules and retain Camk2n1 and Snap25 (with Dynll2), while non-hubs are more diffuse with these synaptic genes weaker. In Cluster 5, hubs bridge to excitatory cells and show Mbp with Tmsb4x, whereas non-hubs stay mostly intra-astrocytic. In Cluster 6, hubs link at the Cluster 2–Cluster 6 interface and express Camk2n1, Ndrg4, App, Slc25a4, Olfm1. Together, hubs preferentially wire within clusters and preserve function-matched programs, while non-hubs mix across clusters and reflect neighbor-aligned expression.

Together, these results show that MAGNET reconstructs spatially organized communication networks with hub cells that are neuron-enriched, exhibit directed cluster preferences, and express gene programs aligned with synaptic signaling and calcium-dependent pathways.

### Attention-based analysis

**Fig 8:**
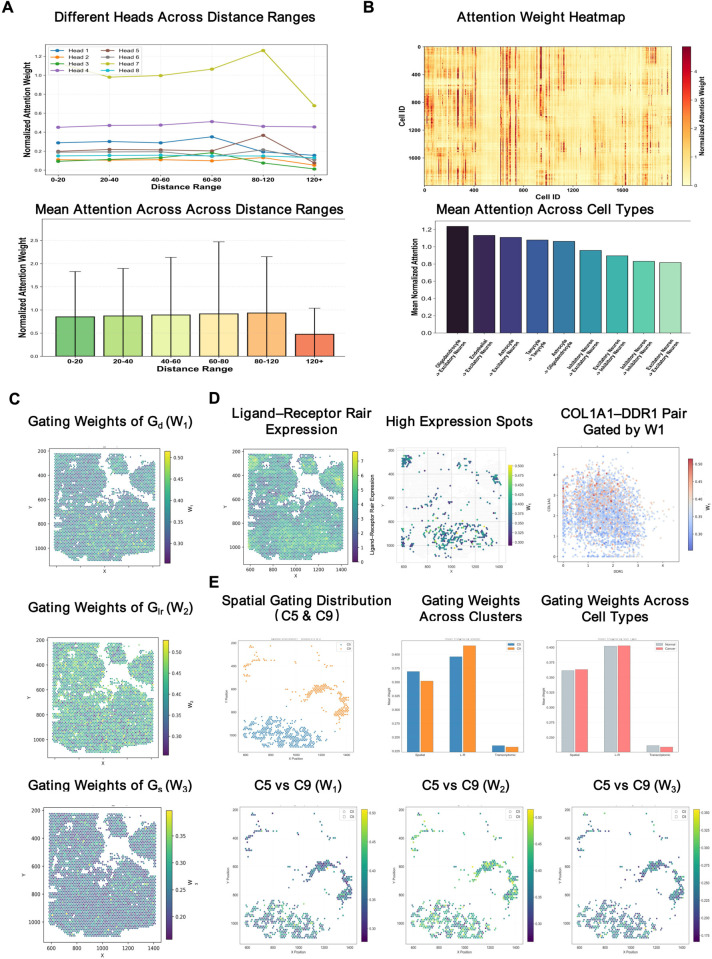
Attention–gating analysis of MAGNET. **(A)** Mean normalized attention per head across spatial distance bins. **(B)** Cell-level heatmap and cell-type summaries showing how attention scores are distributed across cells and across cell types. **(C)** Gating maps for the three views (*W*_1_, *W*_2_, *W*_3_). **(D)** Ligand–receptor expression visuals: spatial map of LR-pair intensity (left), high-expression spots (middle), and an example pair (COL1A1–DDR1) overlaid with spatial gate *W*_1_ (right). **(E)** Cancer-enriched regions focusing on cluster 5 and cluster 9: spatial locations of the two clusters; summaries of *W*_1_, *W*_2_, and *W*_3_ by cluster and by cell type; and spatial maps of *W*_1_, *W*_2_, and *W*_3_ within cluster 5 and cluster 9.

To further probe interpretability, we analyzed MAGNET’s attention and gating outputs (Fig 8). MAGNET integrates three view-specific graphs—spatial proximity (*G*_*d*_), ligand–receptor interaction (*G*_*lr*_), and transcriptional similarity (*G*_*s*_)—via a gated fusion module that assigns a per-cell weight to each view. In Fig 8, the gating maps *W*_1_, *W*_2_, and *W*_3_ visualize these weights and therefore indicate the relative contribution of the spatial, ligand–receptor, and transcriptional channels, respectively; higher *W*_1_ highlights proximity-driven links, higher *W*_2_ emphasizes ligand–receptor signaling, and higher *W*_3_ reflects transcriptional similarity.

First, the multi-head curves show that different heads preferentially attend to distinct spatial distance ranges (Fig 8A), thereby mitigating a trivial bias toward immediate neighbors and enabling the model to capture both local and mid- to long-range interactions. Second, cell-level and cell-type–level summaries show how attention scores are apportioned among cells and among cell types (Fig 8B), revealing context-dependent attention allocation rather than a uniform focus. Third, the gating maps display complementary spatial patterns in which the spatial (*W*_1_), ligand–receptor (*W*_2_), and transcriptional (*W*_3_) views emphasize proximity, signaling, and expression similarity, respectively (Fig 8C), underscoring how the three views contribute to the fused prediction. In Fig 8D, the spatial distribution of ligand–receptor expression intensity is visualized; areas of high LR intensity coincide with larger spatial weights *W*_1_, and *W*_1_ scales positively with ligand intensity. Finally, cancer-enriched regions exhibit heterogeneous *W*_1_–*W*_3_ profiles and spatial distributions (Fig 8E). For example, Cluster 5 (C5) shows stronger spatial gating (*W*_1_), whereas Cluster 9 (C9) shows stronger ligand–receptor gating (*W*_2_).

## Discussion

We present MAGNET, a deep learning framework designed to reconstruct CCI networks. By integrating multi-view graph modeling with a Cell–Gene attention mechanism, MAGNET integrates intercellular communication graphs with gene regulatory network information to uncover functional heterogeneity within cell cluster.

MAGNET demonstrates strong performance across multiple benchmark datasets in predicting interaction networks, with ablation studies underscoring the critical roles of both multi-view integration and the Cell–Gene attention module. Applied to breast cancer spatial transcriptomics data, MAGNET identified two distinct spatial modules: GEM14 and GEM6. GEM14, located at the tumor–normal interface, showed enrichment in antigen-processing and interferon signaling, suggesting epithelial–immune interactions. In contrast, GEM6, prevalent near malignant cells in matrix-rich regions, was associated with receptor tyrosine kinase signaling and matrix remodeling, marked by elevated SPP1 and FN1 expression.

These results demonstrate that MAGNET effectively reconstructs cell–cell interaction networks, where high-scoring edges reflect meaningful functional relationships. While the preliminary functional analysis highlights the potential relevance of the predicted modules, further studies are needed to validate the specific cell–cell interactions underlying these modules, and to experimentally characterize the signaling pathways and ligand–receptor pairs involved.

## Supporting information

S1 Fig Comparison of reconstructed cell–cell interaction networks by MAGNET and the SNF fusion network. (A) Global (left two panels) and regional (right two panels) views of the top 20,000 weighted edges from MAGNET and from the SNF fusion network. (B–E) Representative ligand–receptor subnetworks (Nts–Ntsr1, Oxt–Oxtr, Gal–Galr1, and Tac1–Tacr1) demonstrating that MAGNET recovers more spatially contiguous and biologically plausible communication links across cell types and regions. (F) Quantitative summary for the top 20,000 weighted edges. For each method we report the proportion of LR-supported edges with both ligand and receptor expressed (non-zero expression); the proportion of Nts–Ntsr1–supported edges at high expression; the proportion of Tac1–Tacr1–supported edges at high expression; and the overall proportion of LR-supported edges at high expression. High expression is defined as both genes having Z-scores greater than one in the respective source and target cells. Expression values were Z-scored across cells.
(TIFF)

S2 Fig Computational scalability of MAGNET on the MERFISH dataset. To evaluate the computational scalability of MAGNET, we analyzed its performance on subsets of the MERFISH dataset containing 100, 500, 1000, and 2000 cells. For each subset, gene regulatory networks (GRNs) were constructed using different numbers of genes (20–45). Each row corresponds to a gene-number setting (20, 25, 30, 35, 40, or 45 genes), and each column represents a different evaluation aspect: (left) total training time, (middle) peak memory usage, and (right) model performance (ROC-AUC, AP, and F1 score). Bars denote computational cost, and lines indicate performance metrics. MAGNET exhibited approximately linear increases in runtime and memory with the number of cells (N), while predictive performance remained stable once N ≥ 500 across all gene-number settings. Overall, these results suggest that the model scales well and remains stable as the dataset size increases.
(TIFF)

S3 Fig Robustness of MAGNET to gene selection. To assess the robustness of MAGNET, we evaluated its performance on the MERFISH mouse brain dataset by varying the number of selected genes per cell (20–45) while keeping other parameters unchanged. For each configuration, model performance was evaluated using Average Precision (AP), AUROC, and F1 score. The results show that performance remains stable across gene selection ranges and different subset sizes (100–2000 cells), indicating that MAGNET is robust to moderate changes in hyperparameters and gene selection strategy.
(TIFF)

S4 Fig Robustness of MAGNET to GRN noise. To assess the sensitivity of MAGNET to noise in the gene regulatory network (GRN), Gaussian noise of different magnitudes (0%, 10%, 20%, 30%, 60%, and 90%) was added to the GRN matrices before model training. The left panel shows training curves of test AP over 120 epochs under different noise levels, while the right panel summarizes the final AP scores. MAGNET maintained stable training and high accuracy even under severe noise, indicating robustness to uncertainty or incompleteness in GRN estimation.
(TIFF)

S5 Fig Consistency of MAGNET across random seeds. MAGNET was trained using five random seeds to assess the reproducibility of gene selection and model performance. The figure summarizes the overlap of the top 30 biomarker genes, the distribution of gene categories (overlapping, biomarker-only, and seed-only), and the corresponding test AP scores across seeds. Mean expression profiles and gene compositions are further compared across categories, with pie charts showing their proportions. Overall, model performance remains consistent across seeds, demonstrating robustness to initialization and sampling variability.
(TIFF)

S6 Fig Edge-length characteristics of reconstructed interaction networks (MAGNET vs. TENET). Top: spatial layouts of reconstructed edges for MAGNET (left) and TENET (right). Bottom-left: mean and median edge distances for each method. Bottom-right: minimum and maximum recovered distances. MAGNET recovers a larger maximum edge distance than TENET while maintaining comparable mean and median distances, indicating selective discovery of distant interactions.
(TIFF)

S1 Table Performance of MAGNET under different gene network sources. MAGNET was trained using four alternative gene network, including the original gene regulatory network (GRN), the STRING-db protein–protein interaction (PPI) network, and two random network types (Erdős–Rényi and scale-free). The table reports the average AP scores across three spatial transcriptomics datasets.
(PDF)
