# Supplementary figures and images for: MAGNET: Multi-view graph autoencoder with cell-gene attention for cell interaction network reconstruction from spatial transcriptomics
Source: PLoS Comput Biol. 2025 Dec 15;21(12):e1013810. doi: 10.1371/journal.pcbi.1013810 (PMC12716704; doi:10.1371/journal.pcbi.1013810)

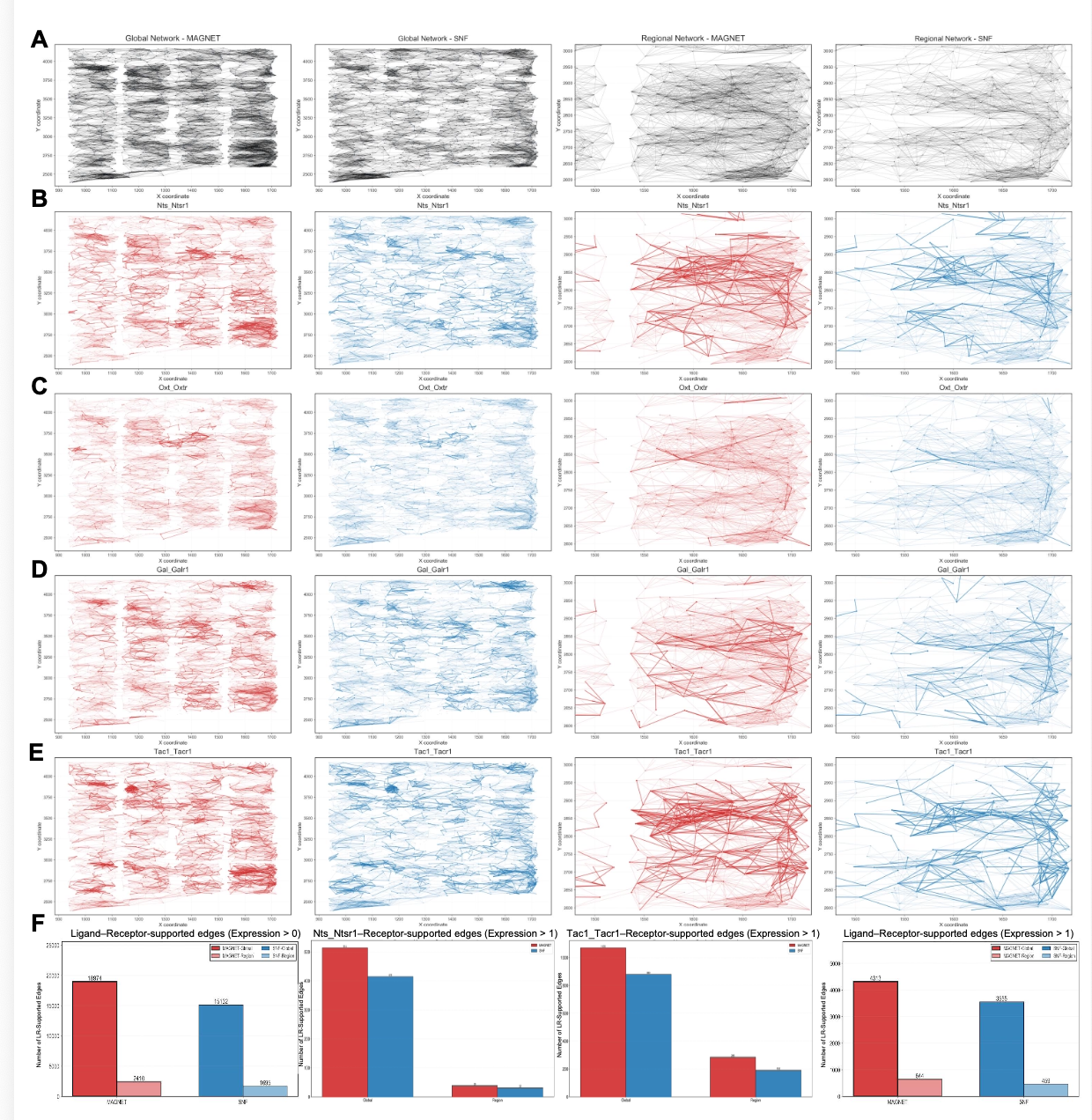

Supplement: S1 Fig — (A) Global (left two panels) and regional (right two panels) views of the top 20,000 weighted edges from MAGNET and from the SNF fusion network. (B–E) Representative ligand–receptor subnetworks (Nts–Ntsr1, Oxt–Oxtr, Gal–Galr1, and Tac1–Tacr1) demonstrating that MAGNET recovers more spatially contiguous and biologically plausible communication links across cell types and regions. (F) Quantitative summary for the top 20,000 weighted edges. For each method we report the proportion of LR-supported edges with both ligand and receptor expressed (non-zero expression); the proportion of Nts–Ntsr1–supported edges at high expression; the proportion of Tac1–Tacr1–supported edges at high expression; and the overall proportion of LR-supported edges at high expression. High expression is defined as both genes having Z-scores greater than one in the respective source and target cells. Expression values were Z-scored across cells. (TIFF) [file pcbi.1013810.s001.tiff]

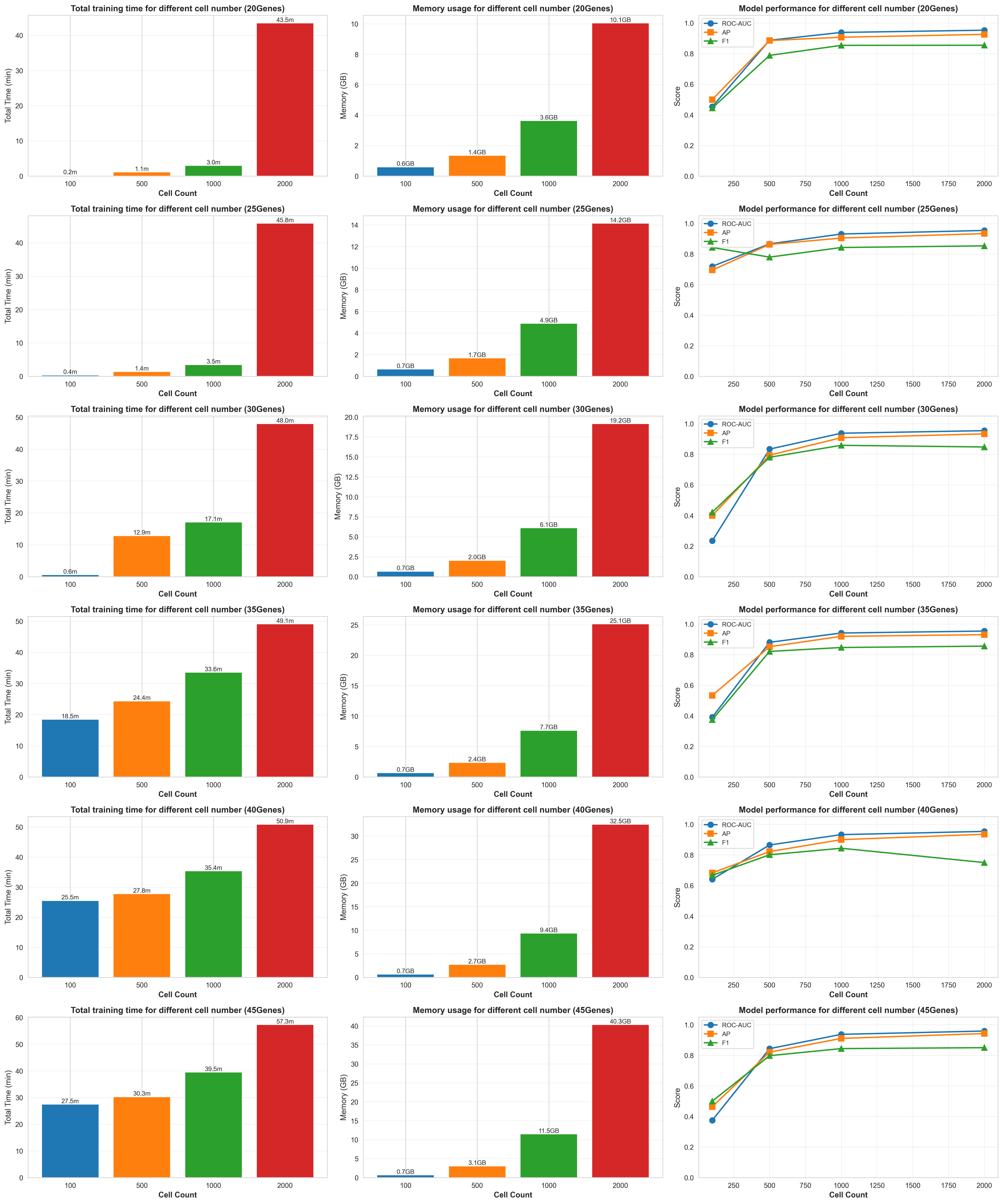

Supplement: S2 Fig — To evaluate the computational scalability of MAGNET, we analyzed its performance on subsets of the MERFISH dataset containing 100, 500, 1000, and 2000 cells. For each subset, gene regulatory networks (GRNs) were constructed using different numbers of genes (20–45). Each row corresponds to a gene-number setting (20, 25, 30, 35, 40, or 45 genes), and each column represents a different evaluation aspect: (left) total training time, (middle) peak memory usage, and (right) model performance (ROC-AUC, AP, and F1 score). Bars denote computational cost, and lines indicate performance metrics. MAGNET exhibited approximately linear increases in runtime and memory with the number of cells (N), while predictive performance remained stable once N ≥ 500 across all gene-number settings. Overall, these results suggest that the model scales well and remains stable as the dataset size increases. (TIFF) [file pcbi.1013810.s002.tiff]

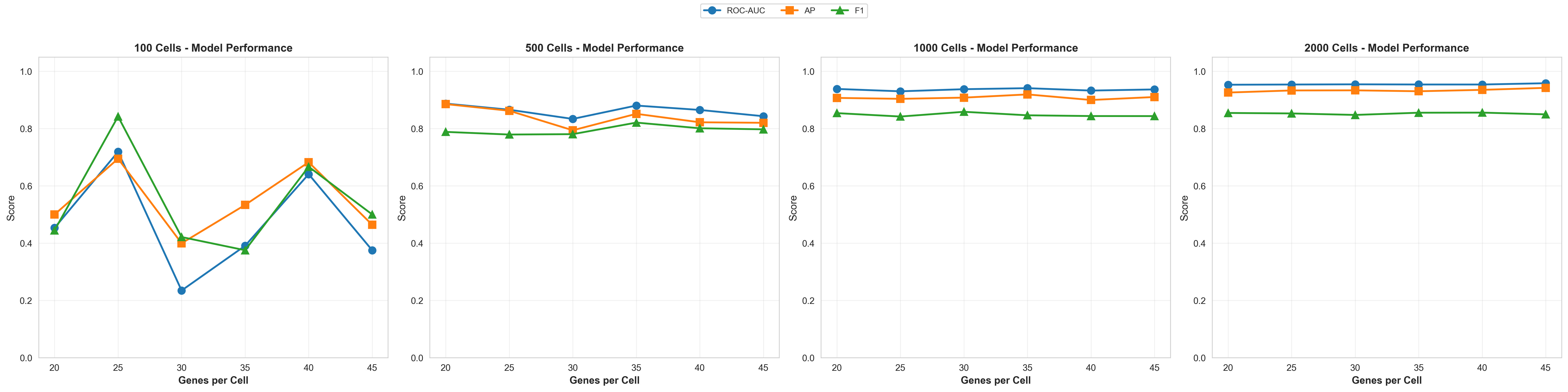

Supplement: S3 Fig — To assess the robustness of MAGNET, we evaluated its performance on the MERFISH mouse brain dataset by varying the number of selected genes per cell (20–45) while keeping other parameters unchanged. For each configuration, model performance was evaluated using Average Precision (AP), AUROC, and F1 score. The results show that performance remains stable across gene selection ranges and different subset sizes (100–2000 cells), indicating that MAGNET is robust to moderate changes in hyperparameters and gene selection strategy. (TIFF) [file pcbi.1013810.s003.tiff]
