# Supplementary material for: MAGNET: Multi-view graph autoencoder with cell-gene attention for cell interaction network reconstruction from spatial transcriptomics
Source: PLoS Comput Biol. 2025 Dec 15;21(12):e1013810. doi: 10.1371/journal.pcbi.1013810 (PMC12716704; doi:10.1371/journal.pcbi.1013810)
Supplement: S1 Table — MAGNET was trained using four alternative gene network, including the original gene regulatory network (GRN), the STRING-db protein–protein interaction (PPI) network, and two random network types (Erdős–Rényi and scale-free). The table reports the average AP scores across three spatial transcriptomics datasets. (PDF) [file pcbi.1013810.s007.pdf]

Network type	seqFISH (AP)	MERFISH (AP)	STARmap (AP)
GRN(CeSpGRN)	0.949	0.932	0.901
STRING-db PPI	0.932	0.916	0.878
Random (ER)	0.826	0.811	0.805
Random (SF)	0.827	0.809	0.816
